# Exploring Caffeine Extraction Using Hydrophobic Deep Eutectic Solvents: Experimental and Theoretical Approaches

**DOI:** 10.1021/acsomega.5c05673

**Published:** 2025-09-20

**Authors:** Khatereh A. Pishro, Leandro S. Silva, Rafaela S. Lamarca, Clarice D. B. Amaral, Mario H. Gonzalez

**Affiliations:** † Department of Chemistry and Environmental Science, 135132 São Paulo State University (UNESP), São José do Rio Preto, São Paulo 15054-000, Brazil; ‡ Department of Chemistry, 28122 Federal University of Paraná, Curitiba, Paraná 81531-980, Brazil

## Abstract

Hydrophobic deep eutectic solvents (HDESs) are emerging as sustainable, biodegradable, and nontoxic alternatives to traditional organic solvents in liquid–liquid extraction processes. This study investigates the use of HDESs, specifically eutectic mixtures of dl-menthol combined with acetic or hexanoic acids, for extracting caffeine from coffee beans (CB), coffee skin (CS), and guaraná drink (GD). Solvent screening using COSMO-RS modeling was performed to identify effective HDES systems. Caffeine detection employed UV/vis spectrophotometry at 274 nm (dl-menthol/acetic acid system) and 284 nm (dl-menthol/hexanoic acid system), with application of Gaussian fitting to minimize spectral interferences. The optimal extraction conditions were determined to be 65 °C and a 1:1 initial solution to solvent (L/L) ratio. Under these conditions, the dl-menthol/acetic acid solvent extracted 0.765 ± 0.007 mg of caffeine per 100 mg of coffee beans, outperforming the dl-menthol/hexanoic acid solvent, which extracted 0.610 ± 0.010 mg. Comparable results were observed for the other matrices, with 0.66 ± 0.01 mg caffeine per 400 mg of coffee skin and 0.57 ± 0.01 mg per 10 mL of guaraná drink. The method presented high precision, with a standard deviation of ±0.0826 mg L^–1^ for five measurements of a 15 mg L^–1^ caffeine solution, and detection and quantification limits of 0.674 and 2.04 mg L^–1^, respectively. The sustainability of the method was evaluated using the AGREE (Analytical GREEnness) metric, obtaining a high greenness score of 0.83, exceeding the values for traditional solvents such as dichloromethane and chloroform. These findings demonstrated the potential of HDESs as greener, safer, and more efficient alternatives for caffeine extraction, aligning with green chemistry principles and supported by both experimental and theoretical evaluations.

## Graphical abstract

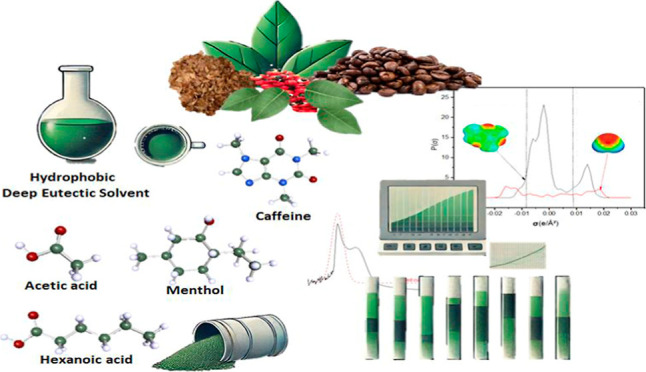

## 1 Introduction

Deep eutectic solvents (DESs) are a novel class of solvents formed by mixing two or more components, typically a hydrogen bond donor (HBD) and a hydrogen bond acceptor (HBA). Unlike traditional solvents, DESs are characterized by low volatility, high thermal stability, biodegradability, and a reduced environmental footprint.^1^ These characteristics have positioned DESs as “green” solvents, making them promising replacements for conventional organic solvents in diverse applications.^2–5^

Recent advances in DES research have introduced hydrophobic deep eutectic solvents (HDESs), which exhibit remarkable solubility for nonpolar compounds. For instance, eutectic mixtures of menthol (a hydrogen bond acceptor derived from natural sources) with fatty acids such as acetic or hexanoic acids (hydrogen bond donors) produce hydrophobic solvents with desirable characteristics. These HDESs present enhanced stability at elevated temperatures, effective catalytic activity, and superior extraction capabilities, as shown in previous studies.^6–10^

The extraction and separation of caffeine remain challenging, often relying on organic solvents such as dichloromethane and chloroform, which are hazardous to both health and the environment.^11^ Although these solvents are effective, they are associated with issues such as toxicity, carcinogenicity, and ozone depletion. Additionally, achieving high extraction efficiencies, while minimizing solvent consumption and waste generation, are critical challenges in caffeine extraction processes. Traditional methods are inconsistent with the principles of green chemistry, highlighting the need for safer and more sustainable alternatives.^12^

Significant advantages of hydrophobic deep eutectic solvents include their low toxicity, biodegradability, and ability to selectively extract nonpolar compounds such as caffeine, so they are ideal candidates for replacing hazardous organic solvents. Their use is in line with the principles of green chemistry, as evidenced by studies reporting high extraction efficiencies and low environmental impacts.^13,14^ For example, recent research has demonstrated the ability of menthol-based HDESs to extract bioactive compounds with efficiencies exceeding 90%, further supporting their use in separation processes.^15^

Caffeine, a widely consumed stimulant found in coffee, tea, and guaraná drinks, has attracted significant scientific attention, due to its solubility properties and health effects. Traditional caffeine extraction methods often use volatile organic solvents such as dichloromethane and chloroform, with the associated ecological and health risks, including carcinogenicity and ozone depletion.^16^ The present work addresses these challenges by introducing HDESs as green solvents that can enhance the sustainability of the caffeine extraction process.

Various techniques have been used to determine the caffeine contents of coffee, common beverages, and sodas.^17^ Among these, UV/vis spectroscopy is easily accessible in most laboratories and is more economical than other methods.^18^ In common with many conjugated organic compounds, caffeine absorbs light at wavelengths ranging from approximately 260 to 274 nm. Conjugation involves the presence of two double bonds, forming a continuous conjugated system, with a single bond allowing the molecule to repeat this pattern multiple times. Applying Beer’s law and analyzing a series of caffeine standards in this absorbance range enables determination of the caffeine content in various substances.^19^

COSMO-RS (Conductor-like Screening Model for Real Solvents) is a quantum chemistry-based method for predicting the thermodynamic properties of fluids and solutions. It calculates the screening charge density on the surface of molecules, which is then used to determine the chemical potential of each species in a solution.^20–22^ This method enables the prediction of properties including activity coefficient, solubility, partition coefficient, vapor pressure, and free energy of solvation. COSMOtherm is a software implementation of the COSMO-RS model, providing a user-friendly interface for performing these calculations.^23^

A notable study employing COSMO-RS for extraction purposes evaluated the effectiveness of various solvents in extracting rubber seed oil, demonstrating the utility of COSMO-RS in estimating solubility and predicting molecular interactions.^24^ In other work, COSMO-RS was used to screen deep eutectic solvents for the extraction of bioactive compounds from Graševina grape pomace,^25^ highlighting its versatility for application in different extraction processes.

Menthol-based HDESs are particularly suitable for this application, since their hydrophobic nature enhances the extraction of nonpolar compounds, such as caffeine.^26^ Their low toxicity and biodegradability are consistent with the principles of green chemistry, providing a sustainable solution for replacing hazardous solvents. Studies have demonstrated the efficacy of menthol-based HDESs in extracting bioactive compounds, with some achieving extraction efficiencies exceeding 90%.^27^

The liquid–liquid extraction (LLE) technique separates compounds based on their solubility in two immiscible liquids, typically water and an organic solvent.^28^ The choice of solvent strongly influences the efficiency of caffeine extraction, with polar solvents such as dichloromethane, chloroform, and ethyl acetate often being favored, due to their effectiveness in solubilizing caffeine, which is a polar compound.^29^ The density difference between the water and organic phases assists their separation, because use of solvents such as dichloromethane increases the density of the bottom layer, resulting in distinct layer formation. For the extraction of caffeine, an aqueous caffeine solution is mixed with an organic solvent, with blending to facilitate movement into the organic phase, and the layers are then separated. Finally, the organic solvent is evaporated to obtain pure caffeine.^30^ This method is widely used because of its efficiency and effectiveness in extracting caffeine from various sources.

In this study, HDESs composed of dl-menthol with acetic or hexanoic acids were used as green and efficient solvents for caffeine extraction. UV/vis spectrophotometry was used to determine the caffeine contents of coffee beans, coffee skin, and guaraná drink. The COSMO-RS model was employed to study solvent screening and interaction energies, while the extraction conditions were optimized using response surface methodology (RSM).^31^ Figure 1 illustrates the quantum mechanical optimization of the chemical structures of several solvents considered for caffeine extraction in this study.

**Figure 1:**
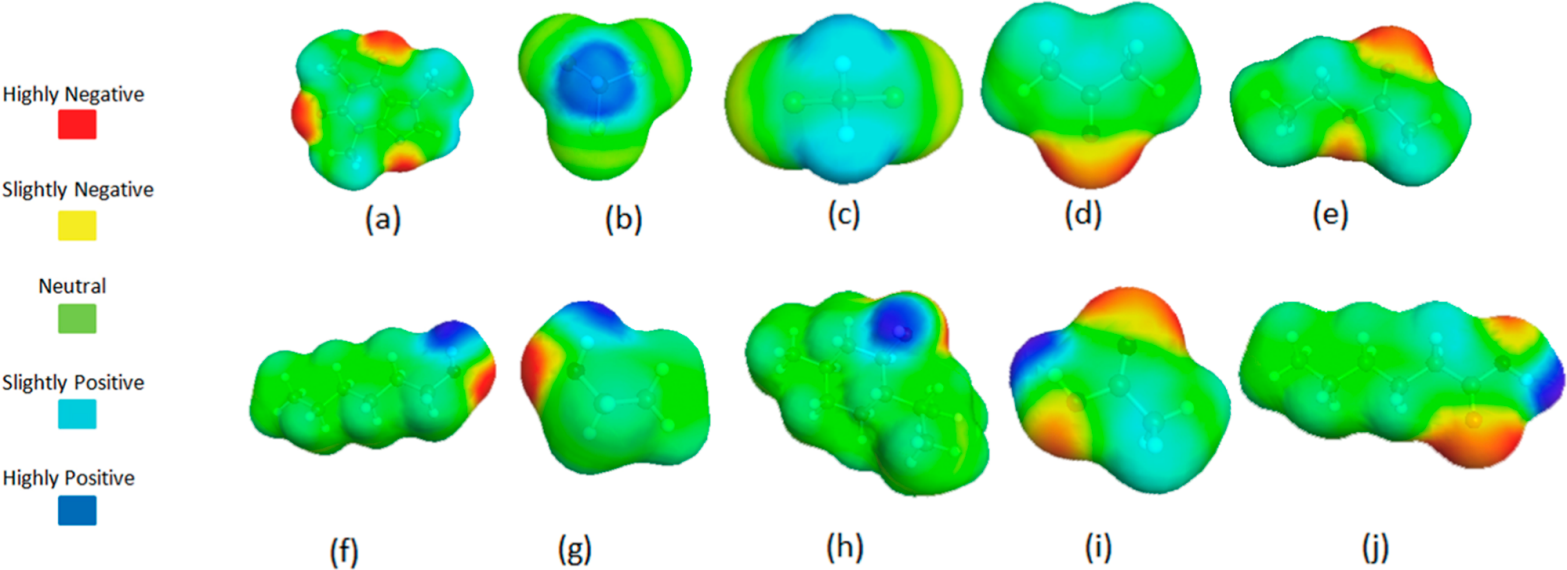
Quantum mechanically optimized chemical structures of caffeine (a), chloroform (b), dichloromethane (c), acetone (d), ethyl acetate (e), hexanol (f), ethanol (g), dl-menthol (h), acetic acid (i), and hexanoic acid (j).

This research combines experimental and theoretical methods to demonstrate how HDESs can replace traditional organic solvents in caffeine extraction. It highlights their potential to make the process more environmentally friendly, without compromising efficiency.

## 2 Materials and Methods

### 2.1 Chemicals

dl-Menthol, hexanoic acid, and acetic acid were acquired from Sigma-Aldrich (Germany). A caffeine standard was also obtained from Sigma-Aldrich. Coffee beans (100% Arabica) were provided by Brazilian suppliers. Coffee skins and guaraná drink were purchased locally in São José do Rio Preto (São Paulo state, Brazil).

### 2.2 Instrumentation and Preparation

#### 2.2.1 Preparation and Characterization of the HDESs

HDES1 was prepared using a eutectic mixture of dl-menthol and hexanoic acid, in a 1:1 molar ratio, with stirring for 2 h at 200 rpm and 60 °C (Model PC-420D Hot plate Stirrer, Labnet, Edison, Mexico). HDES2 (dl-menthol and acetic acid) was prepared as described previously,^32^ but with a longer time of 2 h. Density and viscosity measurements were performed in triplicate, as reported elsewhere.^33^ Density was determined using a pycnometer calibrated using ultrapure water and an analytical balance (model AG200, Gehaka, Brazil). Viscosity measurements were performed with a Cannon-Fenske viscometer, also calibrated with ultrapure water, at a controlled temperature of 25 °C. Infrared spectra were acquired by Fourier transform infrared spectroscopy (ATR-FTIR), using a Bruker VERTEX 70 instrument operating from 4000 to 500 cm^–1^, with spectral resolution of 4 cm^–1^ and 64 scans.

Although both acetic acid and hexanoic acid are liquids at room temperature, their combination with dl-menthol in a 1:1 molar ratio produces a eutectic-like system characterized by strong hydrogen bonding and a depressed melting point, relative to the individual components. This behavior supports classification as a hydrophobic deep eutectic solvent (HDES), consistent with literature definitions. The 1:1 ratio was selected based on COSMO-RS modeling and preliminary experimental screening, which indicated optimal solvent homogeneity, viscosity, and extraction performance. Other ratios (for example, 1:2 and 2:1) were tested, but showed lower stability or efficiency, reinforcing the suitability of the selected composition.

#### 2.2.2 Caffeine Beverage

The roasted and ground coffee was passed through a 200 μm sieve, to ensure a uniform texture. Approximately 100 mg of sieved coffee was added to 50 mL of distilled water, at 70 °C. The mixture was stirred and gently heated for 15 min, using a magnetic stirrer to enhance the extraction.^34^ The 100% Arabica coffee contained 1.1% caffeine,^35,36^ equating to about 22 mg L^–1^ in the test solution. The mixture was filtered through glass paper, to remove particles, followed by centrifugation for 5 min at 5000 rpm and 5 °C. Analysis of the solution was performed by UV–vis absorbance spectrophotometry, using a Shimadzu UV-2600 instrument, with a slit width of 2 nm and a 1 cm quartz cell. The spectrophotometer was connected to a computer running UVprob software.

A similar procedure was used for the coffee skin biomass with significantly lower caffeine content (around 25% that of the beans).^37^ The coffee skins were ground under nitrogen, followed by sieving (200 μm mesh). Approximately 400 mg of the sieved coffee skin were added to 50 mL of distilled water, at 70 °C, followed by slow stirring and heating for 15 min, using a magnetic stirrer.

Caffeine was also extracted from 10 mL of guaraná drink, using the same HDES method. This Brazilian soft drink is obtained from the seeds of the guaraná plant, known for its high caffeine content. Figure 2 shows (a) the coffee skin and the ground and sieved coffee skin, and (b) the guaraná fruit and the guaraná drink used in this research.

**Figure 2:**
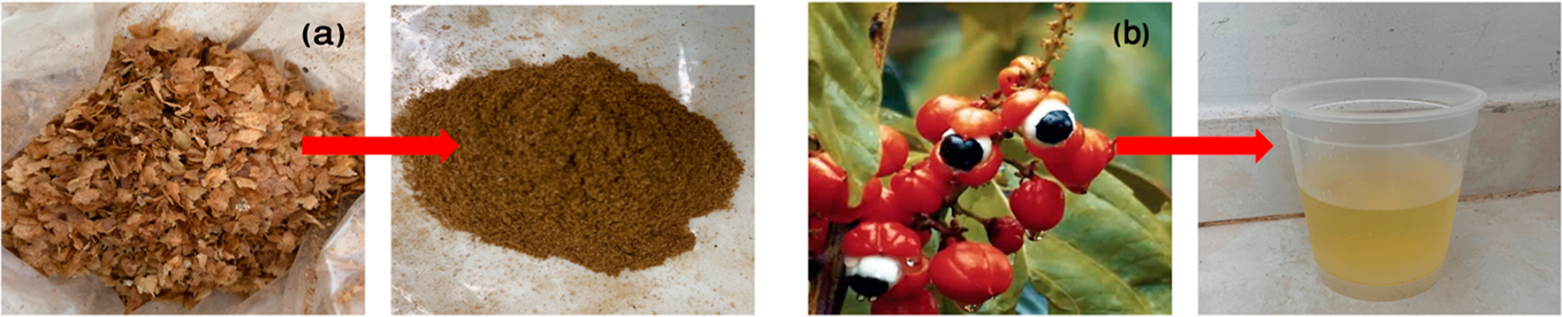
(a) Coffee skin and ground and sieved coffee skin. (b) Guaraná fruits and guaraná drink. Photograph courtesy of Khatereh A. Pishro. Copyright 2025.

The caffeine yields were normalized, based on the sample type. The solid matrices (coffee beans and skin) were dried to constant weight, prior to extraction, and the results were expressed per unit of dry weight. The guaraná drink, as a liquid sample, was analyzed directly and the yield was reported per unit of fresh volume.

Water exhibits poor solubilization ability for caffeine, so it was not used as extraction solvent in this study. Water was the matrix in which caffeine was naturally present or initially dissolved, reflecting realistic sample conditions (such as guaraná drinks and coffee skin infusions). The HDESs formed a separate hydrophobic phase, enabling liquid–liquid extraction driven by favorable partitioning.

#### 2.2.3 Liquid–Liquid Extraction of Caffeine

The coffee solution, described in Section 2.2.2, was mixed with the prepared HDESs (1 and 2) in a 1:1 volume ratio (solution/HDES). Solution/HDES volume ratios of 0.33 and 3 were also tested, to evaluate the effect of solvent amount. The mixtures were homogenized using a magnetic stirrer at different speeds. The resulting solutions were separated and the process was repeated three times, using the same amount of HDES. The HDESs containing the extracted caffeine were collected in separate volumetric flasks for analysis by UV/vis absorbance spectrophotometry. The analyses included HDES reagent blanks. Figure 3 shows a schematic illustration of the experimental setup used for the liquid–liquid extraction of caffeine.

**Figure 3:**
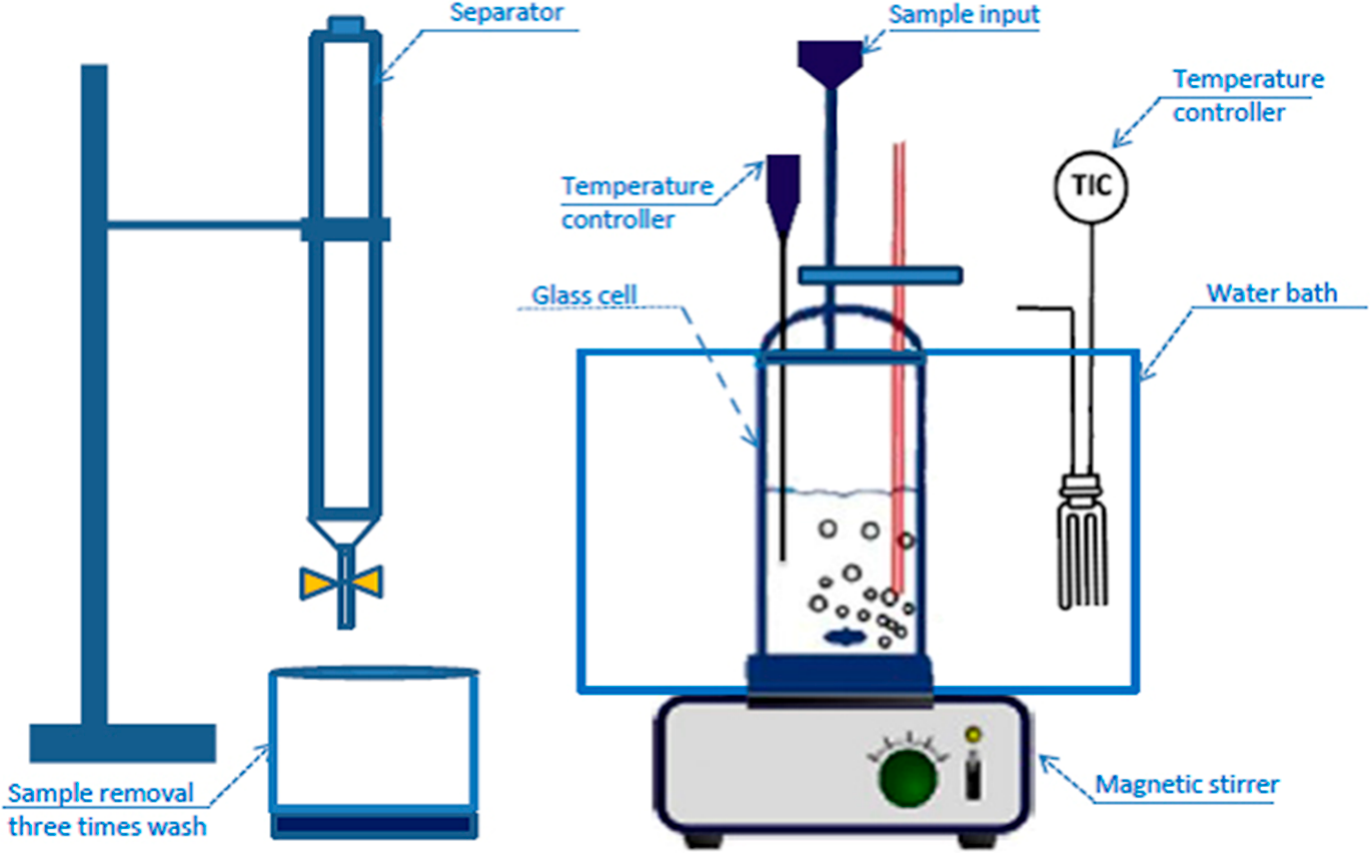
Schematic illustration of the experimental system used for liquid–liquid extraction of caffeine.

#### 2.2.4 Preparation of Standard Solutions, Spectra, and Interfering Bands

A caffeine standard solution (100 mg L^–1^) was prepared by dissolving 10 mg of caffeine powder in 100.0 mL of HDES and stirring for 30 min. Caffeine in HDES1, composed of dl-menthol and hexanoic acid (1:1), was detected at 284 nm, while 274 nm was used for HDES2, composed of dl-menthol and acetic acid (1:1). The absorbance values were plotted against concentrations to create calibration curves.^38,39^

For the caffeine standard in HDES1, only a clear caffeine peak at 284 nm was observed, with no interfering bands (Figure S1). However, when HDES1 was used to extract caffeine from coffee, interfering bands were observed, with peaks at wavelengths in the range from 320 to 350 nm. These peaks probably originated from other compounds extracted from the coffee beans, which influenced the maximum caffeine peak in the spectra (Figure S2). To resolve this problem, a Gaussian function was applied to remove the interference spectra, enabling the peak absorbance to be determined by subtraction of the fitted interference spectra from the overall caffeine spectra. This procedure effectively reduced the influence of the interfering components, enabling more accurate measurements. The same fitting process was applied to the spectra obtained at a wavelength of 274 nm for the analysis of caffeine extracted using HDES2 (dl-menthol/acetic acid, 1:1).

#### 2.2.5 Dynamics of the L/L Extraction Process

The liquid–liquid extraction of caffeine from coffee using HDES2 (menthol/acetic acid, 1:1) was evaluated at 25 and 65 °C, using periods of 2, 5, 10, 15, 20, 25, and 30 min. The extraction efficiencies at the two temperatures were similar, although at 65 °C, equilibrium was reached in around 10 min, while an additional 5 min were required at 25 °C. Rapid attainment of equilibrium is important for minimizing the contact time and enhancing the throughput of the process. Long-term equilibrium results indicated that a significant period of time was needed for stabilization. Tests extending beyond 30 min showed a marked decrease in efficiency at both temperatures, probably due to negative mass transfer effects during the extraction. Figure S3 shows the extraction dynamics at 25 and 65 °C, for analysis at 5 min intervals.

#### 2.2.6 Solvent Screening Using COSMO-RS and Optimization of Conditions

The structures shown in Figure 1 were optimized and the charge density of each compound was calculated using density functional theory (DFT), performed with TmoleX software. After determining the shape and π-profile of each compound, COSMOthermX was employed^21^ with the BP_TZVP_19.ctd parametrization to calculate the logarithm of relative solubility (log_10_(*X*_RS_)) between the solid compound and the liquid solvent.^40^ In addition, a comparison was made of the activity coefficients (ln­(γ)) of different solvents. COSMO-RS was also used to investigate the interaction energies, considering electrostatic misfit energies (*E*_misfit_), hydrogen bond interaction energies (*E*_hb_), and van der Waals energies (*E*_vdW_) between the HDESs and caffeine, according to eqs 4 and 5.^21^ These parameters, combined with the functions in eqs 4 and 5, enabled detailed interaction energy calculations within the COSMO-RS framework.

Statistical optimization of the operational conditions, employing the response surface methodology (RSM) technique, enabled the identification of critical parameters and their interactions. The focus was on three key factors, namely temperature (*T*), time (*t*), and liquid–liquid ratio (L/L), which collectively influenced the response. A second-order polynomial eq (eq 1) was used to model the relationship between the parameters and the response.

1 $$\beta_{0}+\sum \beta_{i}X_{i}+\sum \beta_{ii}X_{i}^{2}+\sum_{i<j}\beta_{ij}X_{i}X_{j}$$

The coefficients β_0_, β_*i*_, β_*ii*_, and β_*ij*_ were considered, where β_0_ represents the intercept term, β_*i*_ corresponds to the linear effect of the independent variable *X*_*i*_, and β_*ii*_ represents the quadratic effect of the independent variable *X*_*i*_. The Python statistical model library was used to fit the model, perform ANOVA, determine the significance of the linear, interaction, and quadratic terms, and evaluate the overall fit of the model (using *R* ^2^). Response surface plots were generated using matplotlib to visualize the effects of temperature and L/L ratio at constant time, L/L ratio and time at constant temperature, and temperature and time at constant L/L ratio. These analyses enabled the identification of significant factors and interaction effects, with the nature of the response surface providing a clearer understanding of the process variables and their influences on the response.

#### 2.2.7 AGREE (Analytical GREEnness) Metric

The HDES-based liquid–liquid extraction method, with UV–vis analysis, was assessed for greenness and safety using the AGREE metric, which is a tool commonly applied to evaluate green analytical methods.^41^ The AGREE score was compared with those for caffeine extraction methods utilizing dichloromethane and chloroform. The score obtained using the AGREE calculator highlighted aspects related to green analytical chemistry (GAC)^42^ and the AGREE framework. These tools constitute a comprehensive and accessible approach to the evaluation of analytical methods, considering all phases, reagents, and instrumentation, representing a recent advancement in the field. Each criterion corresponds to one of the 12 principles of GAC and is scored numerically from 0 (minimum compliance) to 1 (full compliance).

## 3 Results and Discussion

### 3.1 Solvent Screening Using COSMO-RS

#### 3.1.1 Sigma Profiles

The sigma (σ) profiles of compounds are valuable indicators of their chemical characteristics, including polarity and hydrogen bonding capabilities, which can assist in understanding potential interactions between the target solute and solvents during extraction.^21,43^ The sigma profiles of caffeine and water are shown in Figure 4a. The polarized charge distribution (σ) of the caffeine molecule provided insights into its potential interactions with solvents. A moderate peak in the negative σ region, at around −0.005 (e/Å^2^), was indicative of the presence of hydrogen bond donors, probably associated with the nitrogen atoms in the imidazole ring structure of caffeine, which could participate in hydrogen bonding by means of its hydrogen atoms. A significant peak in the central region of the σ profile, at around 0.00 (e/Å^2^), suggested a substantial nonpolar character, attributed to the hydrophobic regions of the caffeine molecule, including the hydrocarbon segments and the planar aromatic ring structures. A notable peak in the positive σ region, at around +0.015 (e/Å^2^), suggested a strong presence of hydrogen bond acceptors, primarily due to the oxygen atoms in the carbonyl groups and the nitrogen atoms in the aromatic rings of caffeine. These atoms have lone pairs of electrons available for hydrogen bonding.^44^ The σ profiles of caffeine and water illustrated their contrasting molecular interaction characteristics. In the case of caffeine, its hydrogen bond donor–acceptor characteristics and hydrophobic regions enable interactions in both polar and nonpolar environments. In contrast, the interaction potential of water is dominated by its strong hydrogen bonding capability, reflecting its behavior as a highly polar solvent. These differences are critical in understanding the solubility, interaction mechanisms, and behavior of caffeine in various environments.^44,45^

**Figure 4:**
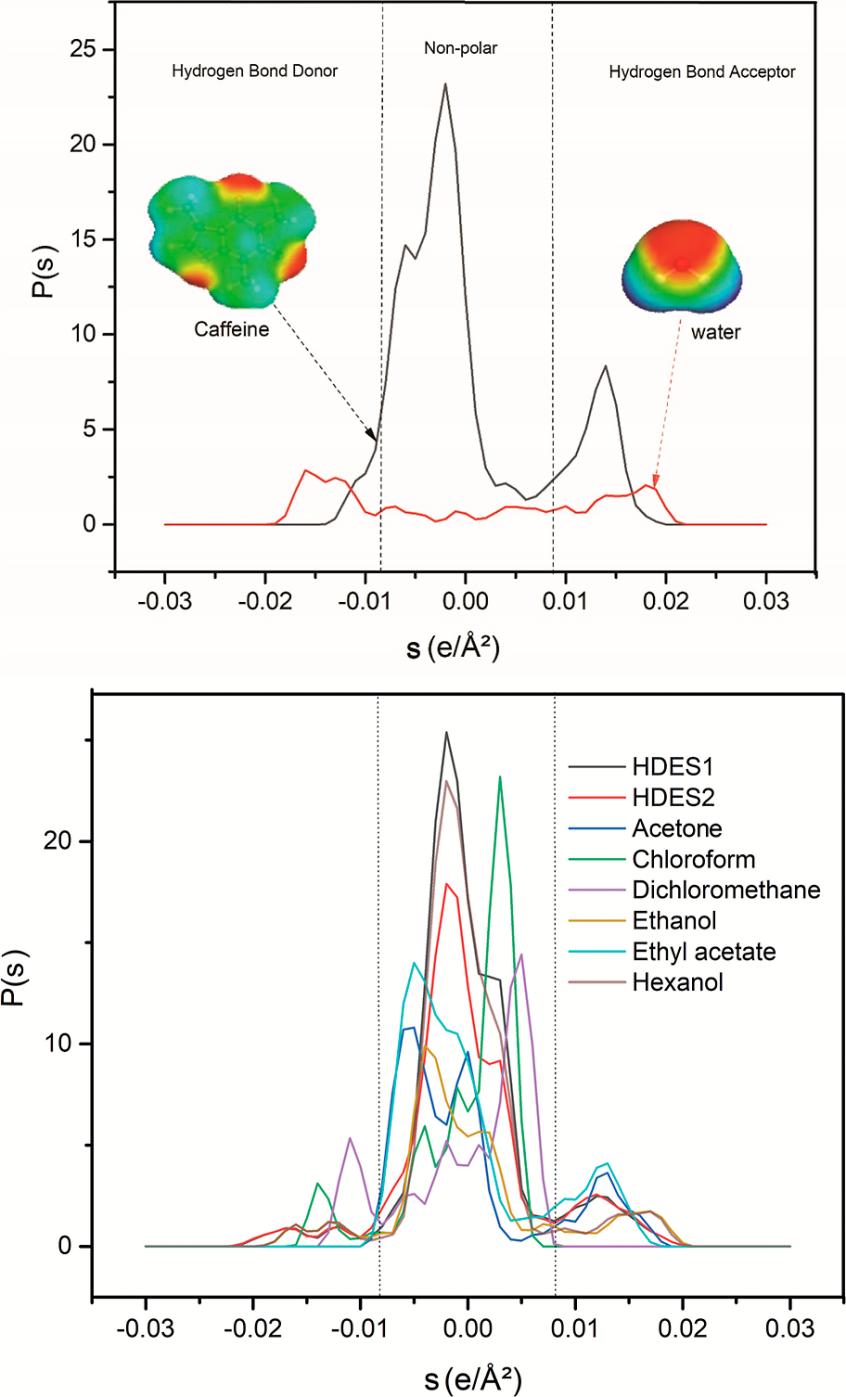
(a) Sigma profiles of caffeine and water, indicating the polar and nonpolar regions and showing the molecular surfaces. (b) Sigma profiles of HDES1 and HDES2, compared to other organic solvents.

Caffeine exhibited a balanced σ profile, with moderate hydrogen bond donor and strong hydrogen bond acceptor capabilities, together with significant nonpolar regions. This profile, with strong hydrogen bond acceptor capability, was indicative of the solubility of caffeine in polar solvents that can act as hydrogen bond donors, such as water and alcohols. On the other hand, the nonpolar regions indicated that caffeine can also interact with nonpolar solvents, although the solubility might be lower, compared to its solubility in polar solvents. Therefore, solvents that have good hydrogen bond donor properties and some nonpolar character could be ideal for dissolving caffeine. Mixtures of polar solvents with nonpolar cosolvents could provide enhanced caffeine solubility and extraction efficiency.^46,47^ The σ profile of caffeine provided a detailed understanding of its chemical properties, highlighting its moderate hydrogen bond donor and strong hydrogen bond acceptor capabilities, as well as significant nonpolar character. These are crucial insights for selecting optimal solvents for the extraction of caffeine and understanding its behavior in different solvent environments.

The σ profiles of HDES1, HDES2, and several organic solvents, shown in Figure 4b, enabled a comparative analysis of their hydrogen bonding and polarity characteristics. HDES1 (dl-menthol/hexanoic acid, 1:1) showed a broader and more negative sigma distribution, indicating higher polarity and stronger hydrogen-bonding potential, compared to HDES2 (dl-menthol/acetic acid, 1:1). The longer hydrophobic chain of the hexanoic acid in HDES1 enhances its interactions with less polar compounds, while its hydrophobicity reduces its ability to dissolve polar solutes such as caffeine. In contrast, HDES2 presented a narrower sigma distribution and lower hydrophobicity, due to the shorter chain of acetic acid, enabling it to outperform HDES1 in solubilizing caffeine. This resulted from specific hydrogen-bonding interactions between acetic acid and the polar functional groups of caffeine, together with the flexibility and smaller size of acetic acid, which allowed better accommodation of caffeine molecules.

Compared to the conventional solvents, HDES1 and HDES2 exhibited broader sigma distributions, bridging the gap between highly polar solvents, such as ethanol, and nonpolar solvents, such as dichloromethane. Ethanol, with a broad and highly negative sigma distribution, provides excellent dissolution of caffeine, due to its strong hydrogen-bonding capabilities. Acetone, with moderate polarity, is also effective, but with lower performance than ethanol. Mildly polar solvents, such as ethyl acetate, are less effective, while nonpolar solvents such as chloroform and dichloromethane show narrow distributions near zero, lacking substantial hydrogen-bonding capacity. Hexanol, which has a long alkyl chain and high hydrophobicity, shows the worst performance, with its molecular characteristics making it unsuitable for dissolving polar solutes.^44,45^ The sigma profile analysis demonstrated that the molecular characteristics of hexanol, dominated by its hydrophobic alkyl chain, make it unsuitable for applications requiring high polarity or strong hydrogen bonding. Solvents with balanced polarity and stronger hydrogen bond donor/acceptor peaks, such as ethanol or acetone, outperform hexanol in such systems.

HDES1 and HDES2 exhibited broader sigma distributions, compared to the conventional solvents, with HDES2 experimentally outperforming HDES1 in solubilization of caffeine, despite its lower polarity. This counterintuitive observation could probably be explained by specific hydrogen-bonding interactions between the acetic acid in HDES2 and the polar functional groups of caffeine, such as carbonyl and amine groups. These interactions are more efficient than those formed by ethanol or acetone.^11^ Furthermore, the smaller and more flexible acetic acid molecule could enhance caffeine accommodation, further increasing solubility.^48,49^ The superior performance of HDES2 in solubilizing caffeine highlighted the importance of specific hydrogen-bonding interactions and molecular flexibility in solvent design. The broader sigma distributions of the HDESs, compared to conventional solvents, indicated their potential for providing tailored solvation behavior.^50–52^ In the case of HDES1, the longer hydrophobic chain of hexanoic acid limited its hydrogen-bonding capacity, consequently reducing its efficiency in dissolving caffeine.

These findings highlighted the potential of the HDESs for tailored solvation applications, with their broad sigma distributions and adaptable polarity making them ideal for systems requiring moderate polarity and hydrophobicity.^11^ This could also explain the superior caffeine solubilization performance of HDES2, despite its lower polarity. The experimental findings revealed the crucial role of specific hydrogen-bonding interactions in caffeine solubility, with the molecular structure of HDES2 resulting in it outperforming HDES1.

#### 3.1.2 Caffeine Solubility and Activity Coefficient

There has been extensive investigation of the solubility of caffeine in organic solvents such as dichloromethane (CH_2_Cl_2_), compared to its solubility in water, with the high solubility in dichloromethane making this solvent preferred for use in liquid–liquid extraction processes.^11^ Evaluation of the solubility of caffeine in the selected solvent is important for obtaining a product with high purity.^29^ In addition, the partition coefficient of caffeine between water and the organic solvent determines the effectiveness of caffeine separation, with a higher partition coefficient favoring the organic solvent being indicative of a more efficient extraction.^53^

The choice of solvent also affects the purity of the extracted caffeine. For example, dichloromethane is effective in separating caffeine from other water-soluble compounds, such as tannins and gallic acid, which remain in the aqueous layer.^16,54^ This efficiency is attributed to the immiscibility of dichloromethane with water and its ability to selectively dissolve caffeine, due to its intermediate polarity. Furthermore, the relatively low boiling point of dichloromethane facilitates its removal after the extraction process, minimizing the risk of residual solvent contamination in the final product. However, the use of dichloromethane raises environmental and health concerns, due to its toxicity and the need for careful disposal, which has led to the exploration of greener alternatives such as ionic liquids, deep eutectic solvents, and other biobased solvents.^16^

The use of hydrophobic deep eutectic solvents (HDESs) for caffeine extraction has both advantages and challenges. These solvents are more environmentally benign, compared to traditional solvents such as dichloromethane, and can make it easier to extract caffeine, while removing impurities including tannins and gallic acid.^26,55^ However, the efficiency of the extraction and the purity of the product depend on the specific HDES used, since their properties can vary widely. One difficulty is that HDESs typically have higher boiling points, making them more difficult to remove completely, which could affect the purity and yield of the caffeine. Despite these challenges, HDESs are biodegradable and less toxic, offering a promising alternative that is in line with the principles of green chemistry.^56–58^ However, there have been no previous studies concerning the liquid–liquid extraction of caffeine from biomass using HDESs, leaving a gap in understanding their full potential and effectiveness in this application.

Quantum chemistry and statistical thermodynamics are used together in COSMO-RS to find the logarithm of relative solubility (log_10_(*X*_RS_)).^29^ This method has been successfully used for the prediction of drug solubility in some solvents, demonstrating its potential in pharmaceutical research.^59^ In this technique, the solute molecule is placed in a virtual conductor and a quantum chemistry simulation determines the surface charge density distribution (σ profile).^60^

In this study, the relative solubilities (log_10_(*X*_RS_)) and activity coefficients (ln­(γ^∞^)) of caffeine in different solvents were calculated at 25 °C, using COSMO-RS, as listed in Table 1. The parameter log_10_(*X*_RS_) is the logarithmic expression of the mole fraction solubility. *X*_RS_ is the mole fraction solubility, representing the solubility of caffeine in the solvent. The solubility data for caffeine in different solvents were consistent with its chemical structure containing both polar and nonpolar regions, as shown in the sigma profile (Section 3.1.1). The highest solubilities were observed using chloroform (*X*_RS_ = 0.355) and acetic acid (*X*_RS_ = 0.224), which are moderately polar solvents. These solvents effectively dissolve caffeine due to their balance of polar and nonpolar properties, allowing strong intermolecular interactions. Dichloromethane, another halogenated solvent with moderate polarity, also showed high caffeine solubility (*X*_RS_ = 0.182).

**Table 1:** Relative Solubility (log_10_(*X*_RS_)), Solubility (*X*_RS_), and Logarithmic Activity Coefficients of Caffeine in Different Solvents at 25 and 65 °C, Calculated Using COSMO-RS

	solubility of caffeine^a^		activity coefficients^b^	
solvents	log_10_(*X*_RS_)	*X*_RS_	ln(γ^∞^) at 25 °C	ln(γ^∞^) at 65 °C
water	–2.690	0.0021	2.2106	3.1159
acetic acid (AA)	–0.650	0.2240	–2.3800	–1.5900
chloroform (Chl)	–0.450	0.3550	–2.7900	–1.4734
dichloromethane (DCM)	–0.740	0.1820	–2.2930	–1.5529
ethanol (Et)	–2.090	0.0081	0.7853	0.4950
hexanol (He)	–2.390	0.0041	1.4728	1.1523
hexanoic acid (HA)	–0.860	0.1380	1.9261	1.1369
ethyl acetate (EA)	–1.860	0.0138	0.2807	0.2304
acetone (Act)	–2.220	0.0057	–0.2404	0.2327
HDES1	2.050	0.0158	0.4658	0.5440
HDES2	–1.640	0.0229	0.8549	0.7674

a The log_10_(*X*_RS_) and *X*_RS_ values are the relative solubility and solubility of caffeine in the solvents at 25 °C.

b The ln­(γ^∞^) values are the natural logarithms of the activity coefficients at infinite dilution (at 25 and 65 °C).

In contrast, water, a highly polar solvent, exhibited the lowest caffeine solubility (*X*_RS_ = 0.0021), because the nonpolar aromatic rings of caffeine lead to low affinity for water. Low solubility was observed in ethanol (*X*_RS_ = 0.0081) and hexanol (*X*_RS_ = 0.0041), which are either weakly polar or have bulky structures that hinder interactions with caffeine. The solubilities were closely aligned with the polarities of the solvents, confirming that superior solubilization of caffeine was achieved for solvents with moderate polarity and low hydrogen-bonding capacity, while highly polar or nonpolar solvents were less effective. This information is essential in applications such as caffeine extraction, where solvents including chloroform and acetic acid are commonly employed.^11^

Although acetic acid showed higher relative solubility of caffeine, based on the COSMO-RS data (*X*_RS_ = 0.2240), its full miscibility with water hinders its utility in liquid–liquid extraction systems. In contrast, the dl-menthol/acetic acid HDES formed a hydrophobic immiscible phase, enabling selective partitioning of caffeine and simplifying solvent recovery. This HDES also had the advantages of reduced volatility, improved handling safety, and potential for reuse, in good agreement with the principles of green chemistry. The experimental results confirmed that the HDES system provided superior extraction performance under biphasic conditions, demonstrating that solute solubility alone does not determine extraction efficiency.^61^

The COSMO-RS model can be used to calculate the chemical potential of a solute (such as caffeine) in different solvents, assisting in determination of the activity coefficients. The relative solubility is calculated by comparing the chemical potential of the solute in each solvent to a reference. This method enables the prediction of solubility and other thermodynamic properties of compounds in a variety of solvents, providing valuable insights for chemical and pharmaceutical research.^21^

Activity coefficients are used in thermodynamics to elucidate deviations from ideal behavior in solutions. In an ideal solution, the interactions between the solute and solvent molecules are uniform. However, in real solutions, these interactions can vary, leading to nonideal behavior. The activity coefficient assists in quantification of this nonideality. The activity coefficient (ln­(γ_*i*_)) was determined using eq 2.^62^

2 $$\ln\left(\gamma_{i}\right)=\frac{{\mu_{i}}^{S}-\mu_{i}E}{RT}$$

where, γ_*i*_is the activity coefficient of solute *i*, μ_*i*_^s^ is the chemical potential of solute *i* in the solvent system, μ_*i*_^E^ is the chemical potential of solute *i* in its pure state, *R* is the universal gas constant, and *T* is the temperature in Kelvin.

Another important concept is the activity coefficient at infinite dilution $\left({\gamma_{i}}^{\infty }\right)$ , represented by eq 3, defined as the activity coefficient when the concentration of the solute *i* approaches zero.

3 $${\gamma_{i}}^{\infty }=\lim_{X_{i}\to 0}\gamma_{i}$$

Activity coefficient values are typically expressed as ln­(γ). Lower logarithmic activity coefficient values suggest higher solubility of caffeine in the solvent and stronger interactions between their molecules.^63^ The caffeine solubility was assessed using the COSMO-RS method, focusing on the infinite dilution logarithmic activity coefficient (ln­(γ^∞^)) predictions for caffeine in various solvents, at 25 °C and 100 kPa (Table 1).

The COSMO-RS methodology was employed to screen 11 solvents, including HDESs, with varying polarities (polar, moderately polar, and nonpolar). A smaller value of ln­(γ) suggests a stronger affinity toward the solvent. For polar and moderately polar solvents, the solute–solvent interactions are governed by dipole–dipole interactions and hydrogen bonding, while van der Waals forces are most important for nonpolar solvents.^64^ As shown in Table 1, the activity coefficients (ln­(γ^∞^)) and relative solubility values (log_10_(*X*_RS_)) for caffeine in the solvents at 25 and 65 °C were consistent and reasonable.^65^ Less polar solvents such as chloroform and dichloromethane showed higher solute solubility and negative ln­(γ^∞^), indicating favorable solute–solvent interactions, while highly polar solvents such as water and ethanol exhibited low solute solubility and positive ln­(γ^∞^), reflecting unfavorable interactions. The decrease of ln­(γ^∞^) with increasing temperature for most of the solvents was consistent with the expected improvement in solute solubility at higher temperature.^65^ Furthermore, the *X*_RS_ values and their logarithmic conversions (log_10_(*X*_RS_)) aligned with the solubility trends, confirming the accuracy of the data.

For HDES1 and HDES2, which exhibited moderate solute solubility, the caffeine activity coefficients (ln­(γ^∞^)) were calculated using COSMO-RS, to evaluate the interaction strengths. For HDES1 (dl-menthol with hexanoic acid), the ln­(γ^∞^) values were 0.4658 at 25 °C and 0.5440 at 65 °C, while for HDES2 (dl-menthol with acetic acid), the values were 0.8549 at 25 °C and 0.7674 at 65 °C. For HDES2, the activity coefficient decreased with increase of the temperature, indicating greater solubility of caffeine in HDES2 at higher temperature. In contrast, HDES1 showed stronger interaction at lower temperatures. These results supported the experimental observation that HDES2 was a better solvent, especially at higher temperatures. Overall, these findings were consistent with the established understanding of the solubility of caffeine in various solvents.^11^

Since water exhibits poor solubility and unfavorable activity coefficients for caffeine, it was not used as the extraction solvent in this study. Instead, water was the matrix in which caffeine was naturally present or initially dissolved, reflecting the conditions of real samples (such as guaraná drinks or coffee skin infusions). The HDESs formed a separate hydrophobic phase, enabling liquid–liquid extraction driven by favorable partitioning. The COSMO-RS data supported this behavior, showing significantly lower activity coefficients for caffeine in HDES1 and HDES2, compared to water, indicating stronger solvation and enhanced extraction efficiency.

#### 3.1.3 Interaction Energies

COSMO-RS calculations were employed to investigate the interaction energies and mechanisms^20^ for extraction of caffeine using the HDESs (Figures 5 and 6), including hydrogen bonding, van der Waals forces, and electrostatic interactions. The overall interactions between the HDESs and the target compound were relatively strong, particularly in relation to hydrogen bonding.

**Figure 5:**
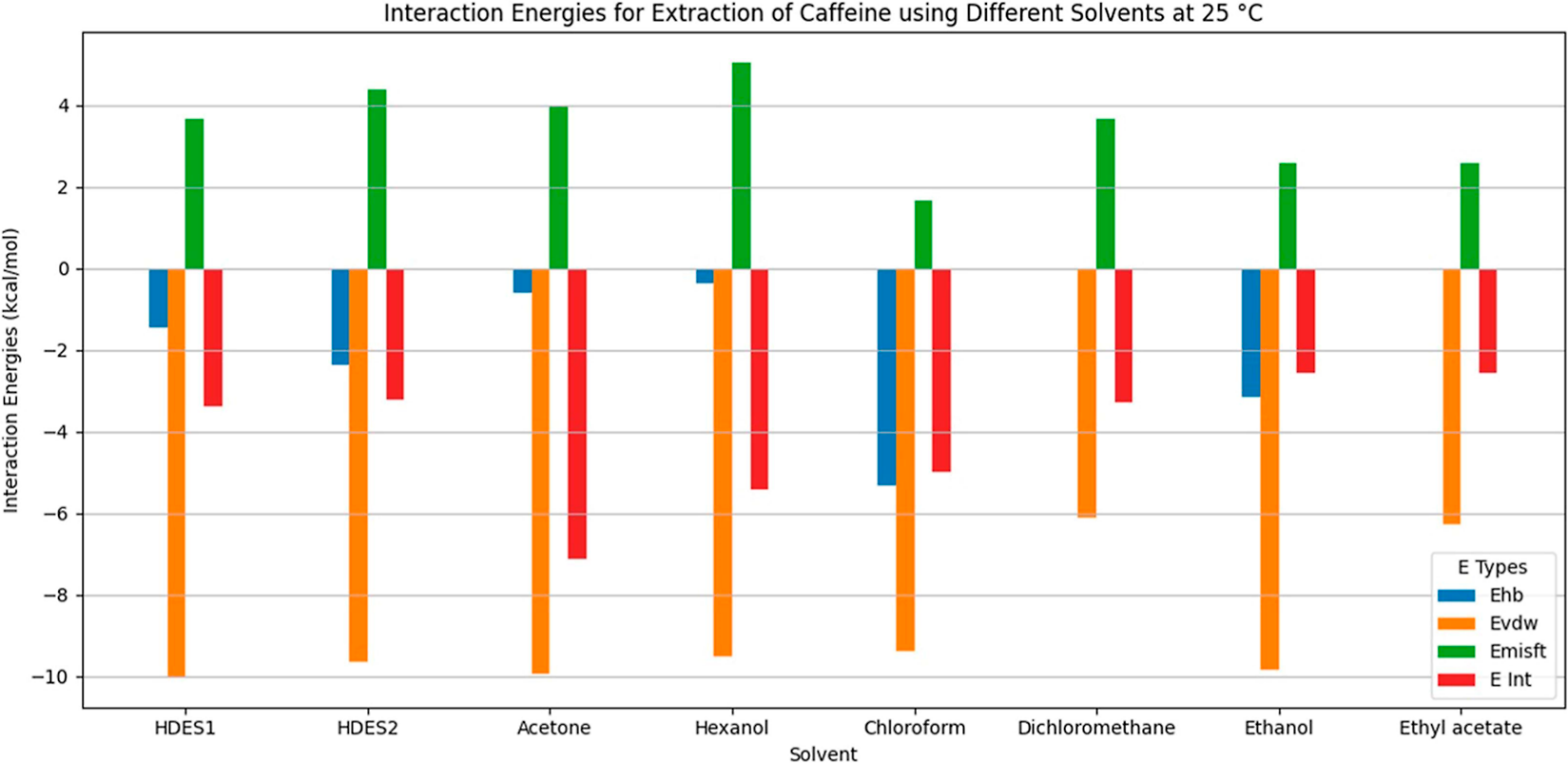
Interaction energies for extraction of caffeine using different solvents at 25 °C.

**Figure 6:**
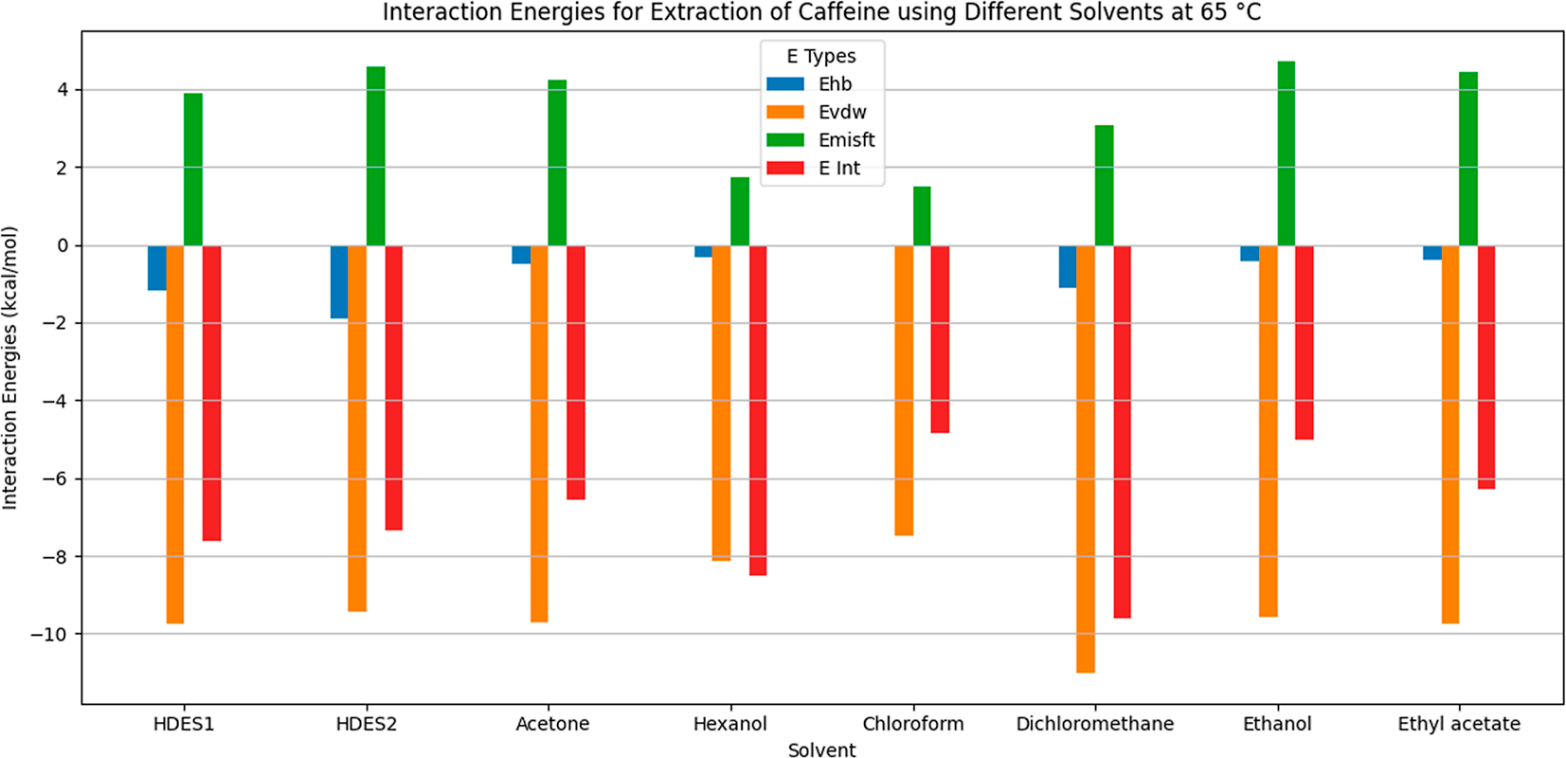
Interaction energies for extraction of caffeine using different solvents at 65 °C.

The free energy of a molecule in a mixture (E_COSMO + dE + Mu)^66^ is a comprehensive measure of how energetically favorable it is for the molecule to reside within the mixture, accounting for internal, differential, and chemical potential energies (see eqs 4 and 5). The total mean interaction energy (H_int) encompasses all forms of molecular interactions, while the misfit interaction energy (H_MF) considers strain due to incompatibility between the solute and solvent molecules. The H-bond interaction energy (H_HB)^67^ indicates stability contributions from hydrogen bonds, while the van der Waals interaction energy (H_vdW)^68^ reflects the influence of van der Waals forces in the mixture. Together, these energies provide a detailed picture of the thermodynamic properties of the system.

Equation for misfit energy

4 $$E_{misfit}\left(\sigma ,\sigma^{\prime }\right)≅a_{cont}e_{misfit}\left(\sigma ,\sigma^{\prime }\right)=\frac{1}{2}a_{cont}a_{misfit}^{\prime }{\left(\sigma +\sigma^{\prime }\right)}^{2}$$

*E*_misfit_(σ,σ^′^) is the misfit energy as a function of the surface charge densities σ and σ′, *a*_cont_ is related to the contact area, *e*_misfit_(σ,σ^′^) represents the misfit energy, *a*_misfit_^′^ is related to misfit energy scaling, and (σ + σ^′^)^2^ is the square of the sum of the surface charge density.

Equation for hydrogen bond (H-bond) energy

5 $$E_{hb}\left(\sigma ,\sigma^{\prime }\right)≅a_{cont}e\left(\sigma ,\sigma^{\prime }\right)=a_{cont}C_{hb}\left(T\right)\min\left(0,\sigma \sigma^{\prime }-\sigma_{hb}^{2}\right)$$

*E*_hb_(σ,σ^′^) is the hydrogen bond energy as a function of the surface charge densities σ and σ^′^, *e*(σ,σ^′^) represents the energy, *C*_hb_(*T*) is a temperature-dependent parameter related to the strength of the hydrogen bond, and *σ*_hb_^2^is related to the critical value for hydrogen bonding.

The evaluation of solubility in a solvent requires consideration of various energy values. The total mean interaction energy (H_int) should be negative, reflecting beneficial overall molecular interactions. The misfit interaction energy (H_MF) should be closer to zero or negative, indicating minimal strain or distortion. The H-bond interaction energy (H_HB) should be more negative, indicating strong and favorable hydrogen bonding, while the van der Waals interaction energy (H_vdW) should also be more negative, showing strong attractive van der Waals forces. Achieving these energy values typically correlates with better solubility of the solute.^24,69^

For HDES1 at 25 °C, the results for the interaction energies in the caffeine, hexanoic acid, and dl-menthol mixture were −1.46528 kcal/mol for hydrogen bonds (*E*_hb_), −9.93852 kcal/mol for van der Waals interactions (*E*_vdw_), and 3.67235 kcal/mol for misfit energy (*E*_misfit_), with a total mean interaction energy (*E*_int_) of −8.31955 kcal/mol. At 65 °C, the hydrogen bond and van der Waals interactions weakened slightly to −1.1916 and −9.75677 kcal/mol, respectively, while the misfit energy increased to 3.90376 kcal/mol, resulting in a total mean interaction energy of −7.63271 kcal/mol. These changes reflected the increased molecular motion and weaker intermolecular interactions at higher temperatures, consistent with the behavior of molecular interactions at different temperatures. Higher temperatures generally result in increased molecular motion, which can weaken specific interactions such as hydrogen bonds and van der Waals forces, which was reflected in the calculated energies.

For HDES2 at 25 °C, the results showed interaction energies of −2.35692 kcal/mol for hydrogen bonds (*E*_hb_), −9.63167 kcal/mol for van der Waals interactions (*E*_vdW_), and 4.37342 kcal/mol for misfit energy (*E*_misfit_). These values contributed to a total mean interaction energy (*E*_int_) of −8.20327 kcal/mol. At 65 °C, the hydrogen bonds became weaker, with an interaction energy of −1.8951 kcal/mol, while values of −9.44599 and 4.57239 kcal/mol were obtained for van der Waals interactions and misfit energy, respectively. This resulted in a total mean interaction energy of −7.35668 kcal/mol. These changes were consistent with the increased molecular motion at higher temperatures, leading to weakened intermolecular interactions.

To determine which HDES was most effective for caffeine extraction, the total interaction energies and their changes with temperature were examined. Better absorption performance is usually suggested by stronger and more negative interaction energies. HDES1 presented interaction energies of −8.31955 kcal/mol at 25 °C and −7.63271 at 65 °C. For HDES2, the values obtained were −8.20327 kcal/mol at 25 °C and −7.35668 at 65 °C. Hence, HDES1 showed slightly stronger interaction energies at both 25 and 65 °C, compared to HDES2. This suggested that HDES1 might be more effective in absorbing caffeine, due to the stronger interaction energies, especially at room temperature. However, the difference was not very large, indicating that HDES1 and HDES2 were close in terms of effectiveness, with slightly higher performance of HDES1.

The interaction energies showed that for both HDES1 and HDES2, the interaction with caffeine was predominantly due to van der Waals forces, with HDES1 showing slightly stronger van der Waals interactions. However, hydrogen bonding was significantly stronger for HDES2, compared to HDES1, indicating that it also made an important contribution to the interaction of HDES2 with caffeine. The misfit energy, indicating general molecular interactions, was higher for HDES2 than HDES1, but was less significant than the other two mechanisms. Overall, van der Waals forces were the dominant interaction mechanism for both HDES1 and HDES2, with a notable contribution of hydrogen bonding for HDES2.

The interactions of caffeine with the other solvents, at both 25 °C (Figure 5) and 65 °C (Figure 6), showed varying contributions of mechanisms including hydrogen bonding, van der Waals forces, and dipole–dipole interactions. In hexanol, hydrogen bonding and van der Waals forces were pronounced at 25 °C, but weakened at 65 °C. Ethanol presented strong hydrogen bonding and dipole interactions, maintaining effectiveness as a solvent at higher temperatures. Chloroform and dichloromethane relied on van der Waals forces and dipole interactions, with reduced solubility of caffeine at higher temperature. For acetone and ethyl acetate, the main mechanisms were dipole–dipole interactions and limited hydrogen bonding, with clear decreases of the interaction energies at 65 °C. Overall, ethanol and acetone were the most effective solvents at the temperatures tested, while the solubility of caffeine in hexanol, chloroform, and dichloromethane decreased at higher temperature, due to weakened interaction energies.

This successful application of the extraction principles validated the experimental hypotheses, while also demonstrating the crucial requirement for accurate and precise measurements in producing reliable scientific data. The results also validated theoretical approaches for solvent selection and the use of RSM for optimization of processes employing different temperatures, times, and concentrations of the solvent used for extraction. Overall, comparison of HDES1 and HDES2 showed that they provided similar performance, in terms of environmental friendliness, chemical properties, and caffeine solubility, due to their analogous compositions. However, considering the lower cost and greater availability of acetic acid, and that the two HDES formulations were roughly equivalent in their interactions with caffeine, HDES2 was selected for use in the subsequent studies, due to its accessibility and practical advantages.

The removal of solvent from coffee is a critical final step in the extraction process. Residual solvents, if not properly removed, can pose significant health risks, due to their potential toxicity, particularly when volatile organic compounds such as chloroform or dichloromethane are used. Furthermore, solvent residues can alter the purity and sensory characteristics of coffee, making it unsuitable for pharmaceutical or food applications. Regulatory standards, such as those established by the International Conference on Harmonization (ICH) and the United States Food and Drug Administration (FDA), provide strict limits on the permissible levels of residual solvents in consumable products, emphasizing the importance of achieving complete solvent removal.^70,71^ From an environmental perspective, recovering and recycling solvents can reduce waste and operational costs, aligning the process with sustainable practices. However, the need for complete solvent removal is less of a concern when using green solvents such as HDESs, since they are nonvolatile, biodegradable, and nontoxic, so any residual solvent remaining in the coffee product poses minimal risk to human health or the environment. This makes HDESs an attractive choice for sustainable and efficient extraction processes, reducing the burden of solvent removal in later stages.^72^

### 3.2 Physicochemical Properties of the HDESs

#### 3.2.1 Density and Viscosity

HDESs are binary or ternary mixtures of compounds, whose formation is driven mainly by hydrogen bonds. HDESs are considered promising alternatives to conventional organic solvents, due to their green and sustainable nature. They exhibit diverse phase behaviors, depending on the forming compounds, molar ratio, and temperature.^73^ Their phase transitions (solid–liquid transitions) are influenced by hydrogen bonding and molecular interactions.

The classification of mixtures based on dl-menthol with acetic or hexanoic acids as hydrophobic deep eutectic solvents (HDESs) is supported by their nonideal mixing behavior, hydrogen bonding interactions, and depressed melting points relative to the pure components. Despite the liquid state of the acids at room temperature, their combination with dl-menthol forms a stable and homogeneous phase with altered physicochemical properties, distinguishing it from a simple solution.^74,75^ Selection of the 1:1 molar ratio was guided by COSMO-RS modeling and experimental screening, which showed that it provided optimal extraction efficiency and solvent stability. Although other ratios were briefly explored, they resulted in phase separation or poorer performance, suggesting that the 1:1 composition was near the eutectic point and was functionally ideal for caffeine extraction. Further studies involving phase diagram mapping and thermal analysis could provide deeper insights into the precise eutectic behavior of these HDES systems.^51^

The densities of HDESs vary depending on their composition, considering the choice of components and their proportions. The presence of water in an HDES results in a notable decrease in density.^76^ In addition, HDESs can have varying viscosities, affecting their flow behaviors. The viscosity decreases with increasing temperature and water content, with greater molecular mobility being associated with reduced viscosity. Essentially, as molecules gain more freedom to move, the overall resistance to flow decreases, leading to a thinner and less viscous substance. This phenomenon is particularly relevant in the case of HDESs, where the tuning of molecular interactions can alter the viscosity.^77^ The density and viscosity of HDES1, containing dl-menthol and hexanoic acid in a 1:1 molar ratio, were 0.971 ± 0.006 g/mL and 9.011 ± 0.044 mPa·s, respectively, at 25 °C. The density and viscosity of HDES2 were reported previously.^32^

In liquid–liquid extraction processes that employ HDESs such as the eutectic mixture of dl-menthol and hexanoic acid, the physicochemical properties of these solvents mean that the viscosity decreases with increase of the temperature, which enhances mass transfer during the extraction, while changes in density influence phase separation and settling of the extracted solute (in this case, caffeine). The presence of water affects both viscosity and density, altering the extraction efficiency.

#### 3.2.2 FT-IR Spectra Analysis

FT-IR spectra provide information about molecular vibrations, with each peak corresponding to a specific bond or functional group. The overall shapes of the spectra for HDES1 and HDES2 were similar, although there were small variations in peak intensities and positions, while specific peaks unique to each spectrum could be attributed to distinct functional groups of the compounds. The FT-IR analysis of HDES2 (dl-menthol/acetic acid, 1:1 molar ratio), together with TG/DTG and DSC analyses, were reported in a previous study.^32^ The FT-IR spectra of HDES1 (dl-menthol/hexanoic acid, 1:1) and its pure components are shown in Figure S4. All three spectra exhibited peaks and troughs indicating the presence of specific functional groups or chemical bonds. Prominent peaks at around 1000–1100 cm^–1^ could be attributed to C–O stretching vibrations. Broad features at around 3000–3500 cm^–1^ could be explained by O–H stretching vibrations (associated with alcohols or carboxylic acids). Peaks near 1700 cm^–1^ were related to ketone or carboxylic acid groups, reflecting the presence of carboxylic acid (hydrogen bond donor).

For exact identification of similarities and differences, it is necessary to have additional context or information about the sample composition. Further analysis, with peak assignments by comparison with reference spectra, would provide additional insights. Nonetheless, the FT-IR results indicated the presence of hydrogen bonds between dl-menthol (hydrogen bond acceptor) and hexanoic acid (hydrogen bond donor), confirming the formation of a new HDES.

Although both acetic acid and hexanoic acid exhibit water solubility, the phases of the HDES systems formed with dl-menthol remained separated and stable under the biphasic extraction conditions used in this study. The elevated temperature (65 °C) maintained the integrity of the HDES phases and prevented precipitation of dl-menthol, which can occur at lower temperatures, due to water-induced disruption of hydrogen bonding networks.^61^ No visible phase inversion or crystallization was observed during or after extraction. FT-IR analysis confirmed the chemical stability of the HDESs, with no significant changes in viscosity or appearance. These results suggested that under controlled conditions, the HDES systems were resilient to mild aqueous exposure and were suitable for short-term reuse. Further studies to assess long-term stability will explore multicycle performance and quantify any leaching effects.

### 3.3 Calibration Curves for Caffeine Standards in the HDESs

The Beer–Lambert law is widely used in many fields, including pharmaceutical science, chemistry, and quantification testing.^78^ The solvent selected to dissolve a compound such as caffeine plays a crucial role in determining its absorption spectrum, because the polarity of the solvent influences the electronic transitions of the solute molecule. Consequently, the effects of different solvents on the energy levels of the electrons can lead to shifts in the absorbance wavelength. Caffeine absorbs UV–visible light due to its conjugated π-electron system, with the maximum absorbance of caffeine in water typically occurring at around 274 nm. In the present work, HDES1 (dl-menthol/hexanoic acid, 1:1) had a polarity that differed from those of water or chloroform,^79^ affecting the electronic transitions of caffeine and resulting in an absorbance shift from 274 to 284 nm (Figures S1 and S2). A red shift (toward longer wavelengths) indicates a less polar environment, while a blue shift (toward shorter wavelengths) indicates a more polar environment. Therefore, the shift to 284 nm indicated that HDES1 was less polar than water. The observed shift was due to a different electronic environment in the π-electron system of caffeine, which could be useful for identifying caffeine in different matrices, or for optimizing analytical methods.^80^

In another study,^81^ a caffeine stock solution (100 mg L^–1^) was prepared by dissolving 0.01 g of recrystallized caffeine in 100 mL of chloroform, in a volumetric flask. Dilutions of 1, 5, 10, 15, 20, and 25 mg L^–1^ were prepared from the caffeine stock solution, followed by measurements of their absorbance at 274 nm in quartz cuvettes (three times for each dilution). A calibration curve for caffeine in the HDES was constructed by measurements of the absorbance at 284 nm for known caffeine concentrations, followed by evaluation of the calibration curves in terms of their linearity and accuracy.

The absorbances of caffeine in water, chloromethane, and HDESs differ, even if the concentration (expressed in parts per million, mg L^–1^) is identical. The solvent in which a compound (such as caffeine) dissolves determines its absorbance spectrum, with the different polarities of solvents influencing the electronic transitions of the molecule.^82^ As discussed above, solvent polarity affects the energy levels of the electrons, leading to shifts in absorbance wavelengths. Water (H_2_O) is a highly polar solvent, due to its ability to form hydrogen bonds. Chloromethane (CH_3_Cl, or methyl chloride) and HDESs are less polar than water. Due to these solvent-specific effects, the absorbance spectrum for caffeine in water may differ from that in chloromethane. The specific wavelengths at which caffeine absorbs light (for example, at around 260 nm) can shift, due to solvent interactions. Consequently, the absorbance (*A*) at a given wavelength may vary, even if the concentration (mg L^–1^) remains constant. Therefore, when using different solvents, it is necessary to measure the absorbance of caffeine solutions in, for example, water, chloromethane, or the prepared HDES, at the same concentration (mg L^–1^). The caffeine remains constant, but the absorbance behavior can vary significantly, depending on the solvent. Hence, careful selection of solvents is needed for optimization of measurements and minimization of solvent-related effects.

Caffeine stock solutions (100 mg L^–1^) were prepared by dissolving 0.01 g of recrystallized caffeine in 100 mL volumes of HDES1 (dl-menthol/hexanoic acid, 1:1) and HDES2 (dl-menthol/acetic acid, 1:1), in volumetric flasks. The caffeine stock solutions were then diluted to prepare concentrations of 5, 10, 15, 20, 25, 30, and 35 mg L^–1^. Absorbance measurements, in triplicate for each dilution, were made in the wavelength range from 200 to 400 nm, with the solutions in quartz cuvettes. The absorbance values were employed to create calibration lines for analysis of the caffeine contents in HDES1 and HDES2. For HDES1, a factor of 15.76 was found by linear regression of concentration against absorbance, so the equation *Y* = 15.76 *X* was used to determine the amounts of caffeine present in the solutions of samples extracted with HDES1. For HDES2, a factor of 17.73 was found by linear regression of concentration against absorbance, so the equation *Y* = 17.73 *X* was used to determine the amounts of caffeine present in the solutions of samples extracted with HDES2.

The method presented satisfactory precision, with a standard deviation of ±0.0826 mg L^–1^ for five measurements of a 15 mg L^–1^ caffeine solution, and limits of detection and quantification (LOD and LOQ) of 0.674 and 2.04 mg L^–1^, respectively.

### 3.4 Optimization of the Extraction Conditions

Liquid–liquid extraction of caffeine from coffee beans, coffee skin, and guaraná drink was performed with the aqueous solutions described in Section 2.2.2, using the experimental system illustrated in Figure 3. The coffee solution was mixed with the solvents (HDESs 1 and 2) in different volume ratios (solution: HDES = 0.33, 1, and 3), at temperatures of 25, 45, and 65 °C. The mixtures were homogenized using a magnetic stirrer at 200 rpm, during contact times from 5 to 15 min. At the end of the contact time, the mixture was transferred to a separator and washed three times with the same volume of caffeine solution. The extracted caffeine in the HDES was collected in a separate volumetric flask and analyzed by measurement of absorbance using a UV/vis spectrophotometer. Analysis of the corresponding HDES reagent blank was included to ensure accuracy of the results. Figure S5 illustrates the mixture of HDES2 (dl-menthol/acetic acid, 1:1 molar ratio) and a caffeine beverage (prepared as described in Section 2.2.2) right after homogenization. Initially, an emulsion formed, but after 15 min, clear phase separation occurred, with the heavier water settling at the bottom and the lighter HDES floating on top.

The extraction process was evaluated using response surface methodology (RSM) to elucidate the effects of temperature (*T*), liquid–liquid ratio (L/L), and time (*t*) on the response variable, by means of a quadratic polynomial model (as described in Section 2.2.6). The procedures were implemented using Python 3.12, with the statsmodels library of Python used to fit the model and perform ANOVA (Table S1), considering the significances of the linear, interaction, and quadratic terms, together with the overall suitability of the model (using *R* ^2^). Figure 7 shows the response surface plots generated using Matplotlib for caffeine extraction using HDES2 (dl-menthol and acetic acid, 1:1), illustrating the effects of time (*t*, min), temperature (*T*, °C), and solvent ratio (L/L). The purpose of these analyses was to identify significant factors, interaction effects, and the nature of the response surface, enabling a clearer understanding of the process variables and their influence on the response.

**Figure 7:**
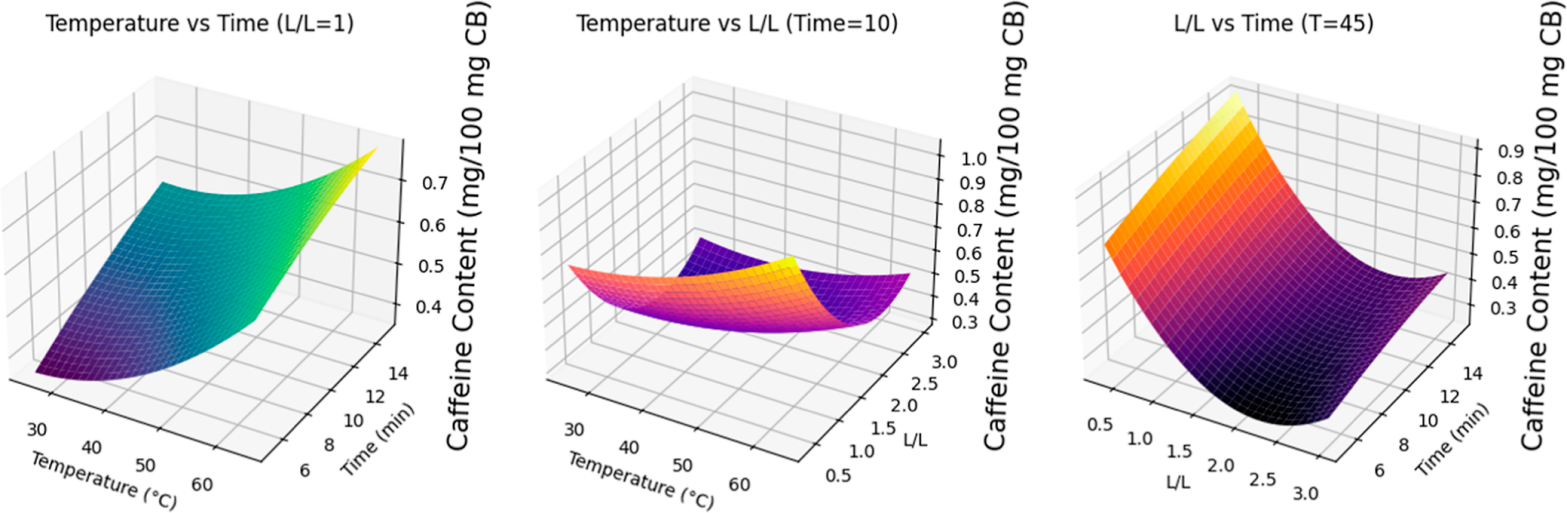
Response surface plots for caffeine content (mg/100 mg CB) using the HDES with dl-menthol and acetic acid (1:1), illustrating the effects of time (*t*, min), temperature (*T*, °C), and solution to HDES (L/L) ratio.

Higher temperatures clearly enhanced caffeine extraction, with the effect becoming more pronounced over longer times, especially at lower L/L ratios. At a fixed L/L ratio, the caffeine content increased with time, with the effect being greater at higher temperature (such as 65 °C). For a fixed time (for example, 10 min), the highest caffeine content was obtained at lower L/L ratios and higher temperatures, while higher L/L ratios led to reduced caffeine extraction. Similarly, at a fixed temperature (for example, 45 °C), lower L/L ratios resulted in a higher caffeine content, with the effect increasing over time. These findings demonstrated that higher temperatures and lower L/L ratios created optimal conditions for caffeine extraction.

The optimized extraction conditions for achieving a high caffeine yield were a temperature of 65 °C, an extraction time of 15 min, and a solution to HDES (L/L) ratio of 1:1. The optimized method using the dl-menthol and acetic acid solvent extracted 0.765 ± 0.007 mg of caffeine per 100 mg of coffee beans (CB), outperforming the dl-menthol and hexanoic acid system, which extracted 0.610 ± 0.010 mg of caffeine per 100 mg of coffee beans. The L/L ratio of 0.33 provided the highest extraction of 1.108 ± 0.008 mg of caffeine per 100 mg of 100% Arabica coffee beans (consistent with high-caffeine Arabica varieties), at 65 °C, with an extraction time of 15 min. However, this condition was approximately 50% more expensive, due to the addition of 50% more solvent (HDES), and would require more energy for solvent removal, compared to the 1:1 ratio. The optimized extraction results are summarized in Table 2.

**Table 2:** Summary of Extraction Results under Optimized Conditions

extraction conditions	caffeine yield (mg/100 mg CB)	observations
65 °C, 15 min, L/L ratio 1:1 (HDES2)	0.765 ± 0.007	the optimized conditions using the dl-menthol/acetic acid solvent (HDES2) led to superior caffeine yield, compared to HDES1.
65 °C, 15 min, L/L ratio 1:1 (HDES1)	0.610 ± 0.010	the caffeine yield using dl-menthol/hexanoic acid (HDES1) was lower than with HDES2, under identical conditions.
65 °C, 15 min, L/L ratio 0.33	1.108 ± 0.008	the highest caffeine yield was achieved, but at the cost of significantly higher solvent usage and energy requirements for recovery.

The method also showed good performance in the extraction of coffee skins (0.66 ± 0.01 mg of caffeine per 400 mg) and guaraná drink (0.566 ± 0.01 mg of caffeine per 10 mL), using the dl-menthol/acetic acid solvent at 65 °C, with an extraction time of 15 min and L/L ratio of 1:1. This demonstrated the adaptability of the method for extracting caffeine from other caffeine-containing sources, in addition to coffee beans. The findings emphasize the need for a balance between extraction efficiency and economic feasibility, with the 1:1 ratio being a practical choice for scalable applications. Further research should explore alternative HDES compositions and energy-efficient solvent recovery methods, with the aim of providing cost-effectiveness, while ensuring satisfactory extraction performance, which could contribute significantly to the development of future sustainable caffeine extraction technologies.

The robustness of the response surface methodology (RSM) model was evaluated using analysis of variance (ANOVA) and regression diagnostics. As detailed in Table S1 (Supporting Information), key model terms such as the linear effect of the initial solution to solvent ratio (L_L, *p* < 0.0001), its quadratic term (I­(L_L^2^), *p* < 0.0001), and the interaction between temperature and L_L (T:L_L, *p* = 0.0098) were statistically significant, indicating strong influences on caffeine extraction efficiency. The model exhibited low residual variance (sum_sq = 0.0632, df = 17) and the regression coefficients confirmed the directionality and magnitude of the contribution of each factor. Nonsignificant terms, such as time and *I*(time^2^), were consistent with the experimental results suggesting minimal impacts, under the tested conditions. These results showed the reliability and predictive accuracy of the second-order polynomial model used for process optimization.

Preliminary assessments of HDES recyclability demonstrated that the dl-menthol/acetic acid system retained over 85% of its original caffeine extraction efficiency after one reuse cycle, with no significant changes in viscosity or phase behavior. The low volatility and thermal stability of the HDES components further supported the potential for solvent recovery and reuse. The use of mild heating and simple separation steps suggested that the extraction method could be readily scaled-up for larger volumes, offering a sustainable and operationally feasible alternative to techniques based on conventional solvents. Future work will focus on multicycle reuse and life-cycle analysis to quantify long-term environmental benefits.

### 3.5 Quantitative Green Chemistry Evaluation

Evaluation of the greenness and safety of the HDES-based liquid–liquid extraction method with UV–vis analysis was performed using the AGREE (Analytical GREEnness) metric (Section 2.2.7). The AGREE score was compared to those for the existing caffeine extraction methods using dichloromethane and chloroform.^41,42^ A graphical representation of the results for the present method is shown in Figure 8, with a final greenness score of 0.83. The evaluation included consideration of the instrument used (a UV–vis spectrophotometer), reagents and HDES components (dl-menthol with acetic acid or hexanoic acid), distilled water use, and energy usage (electricity for UV–vis analysis, heating, stirring, and centrifugation). UV–vis spectrophotometry is a low-energy and nondestructive technique, with minimal waste being generated during the process. The evaluation considered the volume and type of waste, which was mostly water-based and recyclable, including the HDES, filtrates, and paper filters. Assessment of sample preparation included any steps involving heating, filtration, or extraction, with the use of HDES, instead of hazardous solvents such as dichloromethane, providing the benefits of low toxicity and biodegradability.

**Figure 8:**
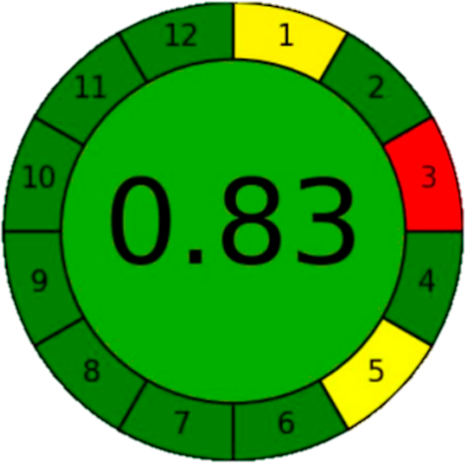
Evaluation of the greenness and safety of the HDES-based liquid–liquid extraction and UV–vis analysis method, using the AGREE (Analytical Greenness) metric.

Based on the input data, the AGREE calculator provides a numerical score based on the 12 principles of green analytical chemistry, where the value (between 0 and 1) indicates the “greenness” of the method. A high score confirms that the method is environmentally sustainable and safe. Specific advantages of the present method, in terms of greenness, were: (1) use of HDES, representing a significant improvement over traditional solvents such as dichloromethane, which are toxic and harmful to the environment; (2) use of UV–vis spectrophotometry, which is energy-efficient, requires no hazardous reagents, and produces minimal waste; and (3) reduced energy consumption, with only mild heating (70 °C) and a short centrifugation step being required, minimizing the environmental impact of the method.

Organic solvents such as chloroform and dichloromethane are highly toxic, with chloroform being a probable human carcinogen and dichloromethane having similar carcinogenic risks, while these volatile organic compounds (VOCs) also contribute to wider environmental pollution.^83^ Their use in caffeine extraction leads to the generation of hazardous waste that is persistent in the environment.^84^ These solvents have low greenness scores, with estimated AGREE values of 0.15–0.25, due to their toxicity, lack of biodegradability, and energy requirements.^37,79^ The Sanofi solvent selection guide^83^ also highlights their environmental and safety risks, recommending safer alternatives such as ethanol or water. For a greener approach, suggested methods include supercritical CO_2_ extraction or solid-phase extraction.^85^ The fundamental principles of green chemistry emphasize the importance of avoiding hazardous solvents, to minimize waste and environmental harm, as discussed in the context of caffeine extraction.^86^

The AGREE score of 0.83 for the present method reflected high greenness, especially when compared to the conventional solvent-based caffeine extraction methods that had significantly lower scores (0.15–0.25). The use of HDES solvents, together with UV–vis spectrophotometry, a low-energy and nondestructive technique, contributed to the overall environmental advantages of the proposed procedure.

The AGREE score of 0.83 reflected the strong alignment of the developed method with the 12 principles of green analytical chemistry. Key contributors to this high score included the use of biodegradable and nontoxic HDESs in place of hazardous solvents, the energy-efficient nature of UV–vis spectrophotometry, and the minimal waste generated during extraction and analysis. The method requires only mild heating and simple sample preparation steps, further reducing the environmental impact. Compared to conventional caffeine extraction methods using dichloromethane or chloroform (AGREE scores ∼0.15 to 0.25), the new approach offers significantly improved sustainability and safety, as illustrated in the AGREE radial plot (Figure 8).

### 3.6 Limitations and Future Work

This study demonstrated that the HDES systems showed promising extraction performance in biphasic aqueous conditions but revealed some limitations. Reuse was restricted to two cycles without regeneration, and when a basic laboratory rotary evaporator hired, the HDES phase retained 85–88% of its caffeine extraction capacity. However, this decline indicates potential instability. While no visible phase separation occurred, changes in composition suggest further analysis is needed. Additionally, the role of water as an antisolvent particularly its ability to leach carboxylic acids and disrupt hydrogen bonding was not quantified. Although operating at elevated temperatures (65 °C) prevented dl-menthol crystallization and preserved phase integrity, the long-term stability of HDES under repeated exposure to aqueous conditions remains uncertain.

Future work will focus on extending HDES reuse through multicycle studies using advanced evaporators, both with and without regeneration. Detailed thermal and compositional analyses (e.g., GC–MS, NMR, FTIR) will be conducted to detect potential degradation or leaching of components. Quantifying water-induced leaching and optimizing HDES formulations for better aqueous resistance and recyclability will also be prioritized. These efforts aim to deepen our understanding of HDES stability and pave the way for the development of robust, reusable solvent systems.

## 4 Conclusions

This study demonstrates the potential of hydrophobic deep eutectic solvents (HDESs) as sustainable and effective options for caffeine extraction. The dl-menthol and acetic acid blend showed superior extraction efficiency, compared to the hexanoic acid variant. The use of UV/vis spectrophotometry enabled precise and economical caffeine quantification, while the COSMO-RS model assisted solvent selection and optimization.

The optimized extraction conditions for caffeine, identified by response surface methodology (RSM), were a temperature of 65 °C, extraction time of 15 min, and solution to HDES (L/L) ratio of 1:1 (using the dl-menthol/acetic acid HDES). These conditions balanced high caffeine yield (0.765 ± 0.007 mg/100 mg CB) with cost-efficiency and enabled high levels of caffeine to be obtained from coffee beans, coffee skin, and guaraná drink, under mild and environmentally friendly conditions. Analysis using the AGREE metric resulted in a high green chemistry score (0.83) for the HDES-based extraction, outperforming traditional solvents such as dichloromethane and chloroform in terms of environmental and safety aspects.

The findings validated the use of HDESs not only for the extraction of caffeine but also as a greener alternative to conventional solvents, aligning with the principles of green chemistry by minimizing ecological and health risks. This research paves the way for further investigations of HDESs in the extraction of other bioactive compounds, supporting their potential use in the food, pharmaceutical, and chemical industries. By advancing both sustainability and efficiency, this study contributes to the development of greener practices in analytical and industrial chemistry.

Despite these promising results, certain limitations were observed. The high viscosity and phase behavior of HDESs may lead to processing challenges in large-scale applications. Additionally, their low volatility could hinder efficient solvent recovery and recycling, potentially affecting long-term sustainability and cost. UV–vis spectrophotometry, despite being economical and low-energy, might not offer the sensitivity or selectivity of chromatographic techniques when analyzing complex matrices.

## Supplementary Material



## Notes

Detailed statistical analysis of the caffeine extraction process, including Table S1 with ANOVA results and regression coefficients derived from response surface modeling; Figures S1 and S2 presenting UV/vis spectra of caffeine standards and extracts obtained using HDES1 (dl-menthol: hexanoic acid, 1:1); Figure S3 illustrating dynamic liquid–liquid extraction profiles obtained with HDES2 (dl-menthol: acetic acid, 1:1) at 25 and 65 °C; Figure S4 showing FT-IR spectra of HDES mixtures and their individual components (dl-menthol and hexanoic acid) (PDF)
