# Supplementary material for: Exploring Caffeine Extraction Using Hydrophobic Deep Eutectic Solvents: Experimental and Theoretical Approaches
Source: ACS Omega. 2025 Sep 20;10(38):44218–33. doi: 10.1021/acsomega.5c05673 (PMC12489721; doi:10.1021/acsomega.5c05673)
Supplement: Supplementary file 1 [file ao5c05673_si_001.pdf]

Supporting Information

Exploring Caffeine Extraction Using Hydrophobic Deep Eutectic Solvents: Experimental and Theoretical Approaches

Khatereh A. Pishro †, Leandro S. Silva †, Rafaela S. Lamarca †,

Clarice D. B. Amaral ‡, Mario H. Gonzalez†*

† Department of Chemistry and Environmental Science, São Paulo State University (UNESP), São José do Rio Preto, São Paulo, 15054-000, Brazil

‡ Department of Chemistry, Federal University of Paraná, Curitiba, Paraná 81531-980, Brazil

* Corresponding author

E-mail address: mario.gonzalez@unesp.br; Phone: +55 17 32212512. Fax: +55 17 32212512.

Tables

Table S1. ANOVA and Regression Coefficients for the caffeine extraction obtained from the RSM (second-order polynomial model).

ANOVA data

	sum_sq	df	F	PR(>F)
T	0.010449	1.0	2.809586	1.119946e-01
L_L	0.321947	1.0	86.569092	4.407940e-08
time	0.006003	1.0	1.614146	2.210228e-01
I(T ** 2)	0.026534	1.0	7.134651	1.611825e-02
I(L_L ** 2)	0.199103	1.0	53.537249	1.203556e-06
I(time ** 2)	0.000840	1.0	0.225914	6.406197e-01
T:L_L	0.031452	1.0	8.457242	9.792269e-03
T:time	0.003046	1.0	0.819167	3.780724e-01
L_L:time	0.006002	1.0	1.613977	2.210460e-01
Residual	0.063222	17.0	NaN	NaN

Regression Coefficients

Intercept	0.668180
T	-0.005213
L_L	-0.518896
time	0.037384
I(T ** 2)	0.000166
I(L_L ** 2)	0.141454
I(time ** 2)	-0.000473
T:L_L	-0.001843
T:time	-0.000159
L_L:time	-0.003220
dtype: float64	

Figures

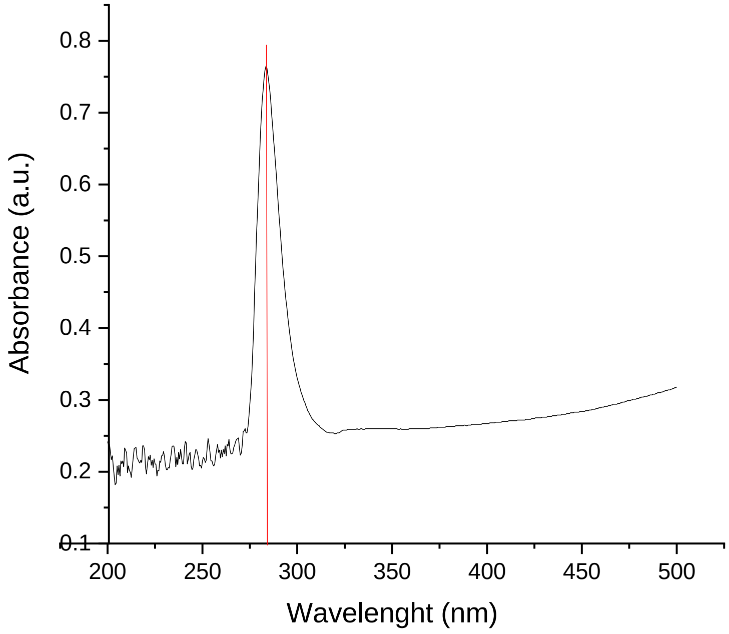

Figure S1. UV–vis spectra of caffeine standard in HDES1 (DL-menthol/hexanoic acid, 1:1).

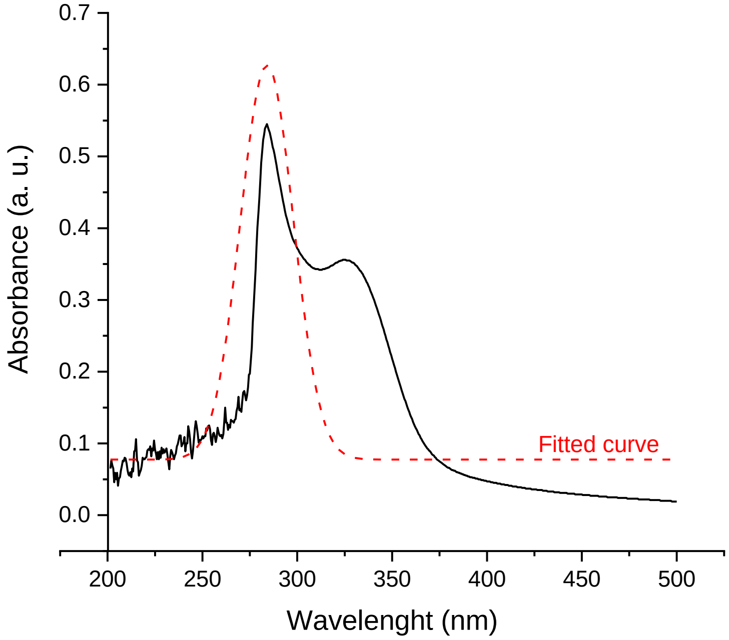

Figure S2. UV–vis spectra of caffeine extracted from coffee beans HDES1 (DL-menthol/hexanoic acid, 1:1).

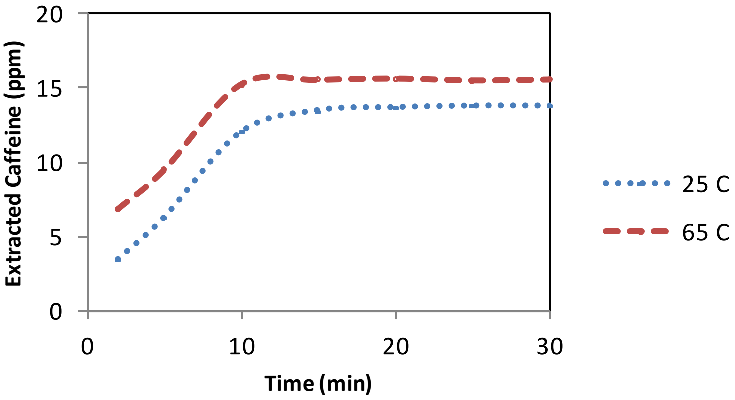

Figure S3. Dynamic liquid-liquid extraction of caffeine using HDES2 (DL-menthol/acetic acid, 1:1) at temperatures of 25 °C and 65 °C.

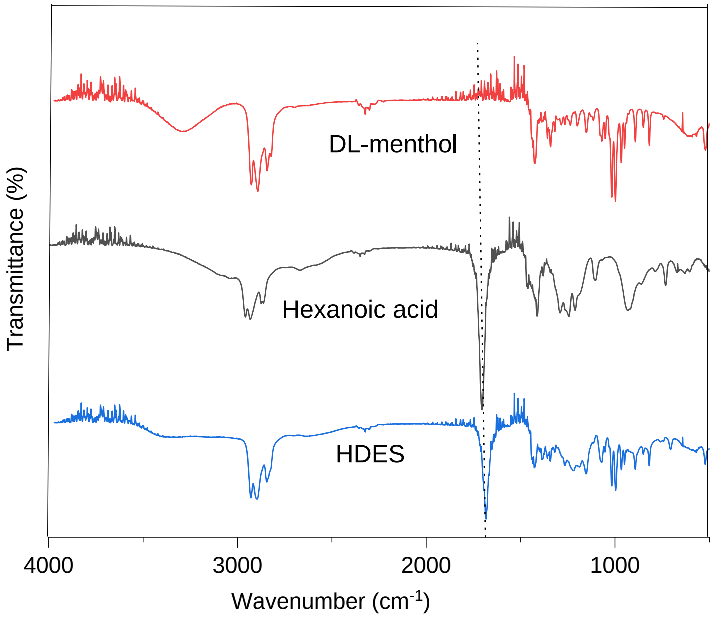

Figure S4. FT-IR spectra's of HDES1 (DL-menthol/hexanoic acid, 1:1), pure DL-menthol and pure hexanoic acid.

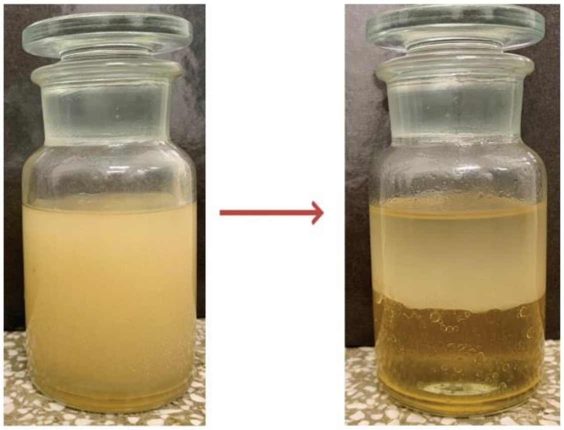

Figure S5. HDES2 (DL-menthol/acetic acid, 1:1) and caffeine beverage mixture immediately and 15 minutes after homogenization.
